# Structural and Functional Analysis of a Bidirectional Promoter from *Gossypium hirsutum* in *Arabidopsis*

**DOI:** 10.3390/ijms19113291

**Published:** 2018-10-23

**Authors:** Jiangtao Yang, Xujing Wang, Agula Hasi, Zhixing Wang

**Affiliations:** 1 Biotechnology Research Institute, Chinese Academy Agricultural Sciences, MOA Key Laboratory on Safety Assessment (Molecular) of Agri-GMO, Beijing 100081, China; jt_y1990@163.com (J.Y.); xujingwang0514@126.com (X.W.); 2 Inner Mongolia Key Laboratory of Herbage & Endemic Crop Biotechnology/College of Life Sciences, Inner Mongolia University, Hohhot 010021, China

**Keywords:** bidirectional promoter, *Gossypium hirsutum*, cloning, transient expression, stable expression

## Abstract

Stacked traits have become an important trend in the current development of genomically modified crops. The bidirectional promoter can not only prevent the co-suppression of multigene expression, but also increase the efficiency of the cultivation of transgenic plants with multigenes. In *Gossypium hirsutum*, *Ghrack1* and *Ghuhrf1* are head-to-head gene pairs located on chromosome D09. We cloned the 1429-bp intergenic region between the *Ghrack1* and *Ghuhrf1* genes from *Gossypium hirsutum*. The cloned DNA fragment GhZU had the characteristics of a bidirectional promoter, with 38.7% G+C content, three CpG islands and no TATA-box. Using *gfp* and *gus* as reporter genes, a series of expression vectors were constructed into young leaves of tobacco. The histochemical GUS (Beta-glucuronidase) assay and GFP (green fluorescence protein) detection results indicated that GhZU could drive the expression of the reporter genes *gus* and *gfp* simultaneously in both orientations. Furthermore, we transformed the expression vectors into *Arabidopsis* and found that GUS was concentrated at vigorous growth sites, such as the leaf tip, the base of the leaves and pod, and the stigma. GFP was also mainly expressed in the epidermis of young leaves. In summary, we determined that the intergenic region GhZU was an orientation-dependent bidirectional promoter, and this is the first report on the bidirectional promoter from *Gossypium hirsutum*. Our findings in this study are likely to enhance understanding on the regulatory mechanisms of plant bidirectional promoters.

## 1. Introduction

Transgenic plants with stacked traits harboring two or more foreign genes can meet the diverse needs of growers and provide multiple benefits; thus, stacked traits have become an important trend in the current development of genomically modified crops. In 2017, the planting area devoted to stacked traits was 77.8 million hectares and covered 41% of the biotech crop planting area globally, representing a 2.5% increase in planting area compared with that in 2016 [1]. 

Co-transformation is an effective strategy for the cultivation of transgenic plants with stacked traits. The selection of a promoter is an important factor for the successful expression of multigenes during co-transformation [2]. Multi-gene engineering strategies are often hampered by sub-optimal expression levels or improper tissue-specificity of particular promoters, or rely on the use of multiple copies of the same promoter, which can result in DNA instability or transgene silencing [3]. A homology of 90 bp in promoter sequences has been reported to be sufficient for the co-suppression of gene expression and transgene silencing [4]. The use of a bidirectional promoter can avoid this disadvantage and increase the efficiency of co-transformation.

A bidirectional promoter is the intergenic region between divergent or head-to-head gene pairs, and bidirectional promoters can drive the simultaneous transcription of divergent gene pairs. With the development of sequencing technology and genome-wide annotations, it has been shown that divergent or head-to-head gene pairs are very common in eukaryotes [5,6]. In the human genome, more than 10% of the genes are arranged in a head-to-head manner with an intervening sequence of less than 1000 bp, and these gene pairs are driven by 1352 bidirectional promoters [5,7,8,9]. However, in the plant genome, researchers have found many head-to-head genes with an intervening sequence of more than 1000 bp [10,11,12]. In the *Arabidopsis* genome, a large proportion (13.3%) of bidirectional gene pairs have been observed, and some of these pairs share an intergenic region of 1 to 1.5 kb [13]. Using the same selection standards as those used for human bidirectional promoters (length less than 1 kb), 8742, 5763, and 8823 divergent gene pairs in total have been identified, accounting for 30.9%, 24%, and 39% of the genomes of rice, *Arabidopsis thaliana*, and *Populus*, and they have confirmed 2106, 1242, and 613 bidirectional promoters in rice, *Arabidopsis*, and *Populus*, respectively [14,15].

In plants, since Shwarz et al. [16] reported the first bidirectional promoter of *maize* chloroplast genes, several bidirectional promoters have been reported in maize, hot pepper, *Arabidopsis thaliana*, rice, soybean, and *Populus* [11,15,17,18,19]. In 2003, Shin et al. identified that the *CaTin 1* and *CaTin1-2* genes in hot pepper had an opposite transcriptional direction and that a 955-bp DNA sequence between the two genes could drive *gus* gene expression in tobacco from two directions, and also regulate the expression of the two genes in response to biotic stress due to pathogen infection, representing the first endogenous bidirectional promoter identified in plants [17]. In analyses of *Arabidopsis thaliana* genomic data, Banerjee found that the *At4g35987* and *At4g35985* genes were bidirectionally transcribed gene pairs, and cloned a sequence of 1258 bp between the two adjacent genes. The authors connected the *gus* and *gfp* genes at both orientations to transform *Arabidopsis thaliana* and tobacco, respectively. The functional verification of the GUS and GFP proteins was performed in both *Arabidopsis thaliana* and tobacco, confirming that the sequence was indeed a bidirectional promoter [11]. In *Arabidopsis thaliana*, the caseinolytic protease B-cytoplasmic (ClpB-C)/heat shock protein 100 protein (AtClpB-C) gene (*At1g74310*) and the choline kinase (AtCK2) gene (*At1g74320*) are located in divergent orientations, and their 1329 bp serves as a heat-inducible bidirectional promoter [19]. In 2014, Liu et al. conducted a genome-wide search in *maize* using genome sequencing results from the inbred line, B73. In total, 1696 bidirectional transcript pairs were identified using a modified search model. The authors functionally characterized the promoter activity of the intergenic regions of most of the bidirectional transcript pairs that were expressed in embryos using a maize embryo transient expression system [18]. In 2015, Liu et al. used the promoter::GUS transgenic approach and revealed that the intergenic region of the *Arabidopsis thaliana* divergent genes *At1g71850* and *At1g71860* was an asymmetric bidirectional promoter, exhibiting an orientation-dependent expression profile. The authors demonstrated that the activity of the At1g71850-At1g71860 bidirectional promoter was modulated by complex interactions between both positive and negative cis-acting elements [20]. In 2016, Wang et al. initiatively combined RNA-seq data and cDNA microarray data to discover the potential bidirectional promoters in rice genomes. Based on the expression level and correlation of each adjacent and oppositely transcribed gene pair, they selected four candidate gene pairs, and subsequently found that GUS and GFP assays of the transgenic plants indicated that all the intergenic regions showed bidirectional expression activity in various tissues of rice [12]. However, to date, there are no reports of a bidirectional promoter from *Gossypium hirsutum*.

In this paper, we report the isolation and characterization of an intergenic region (1429 bp) shared by *Ghrack1* (receptor for activated C kinase 1, Gohir.D09G171000.1) and *Ghuhrf1* (E3 ubiquitin-protein ligase gene, Gohir.D09G171100.1) divergent genes on chromosome D09 from *Gossypium hirsutum.* The transient expression analysis showed that the intergenic sequence could simultaneously drive the expression of the reporter genes *gus* and *gfp* from the forward and reverse orientations. Subsequently, we transformed *Arabidopsis thaliana* to achieve a stable expression, which fully demonstrated that the sequence had bidirectional promoter activity, and was an orientation-dependent bidirectional promoter in *Arabidopsis thaliana*. This study provides the first report of a bidirectional promoter from *Gossypium hirsutum* in *Arabidopsis thaliana*.

## 2. Results

### 2.1. Genomic Organization of the Head-to-Head Gene Pairs, Ghrack1 and Ghuhrf1

**Figure 1:**
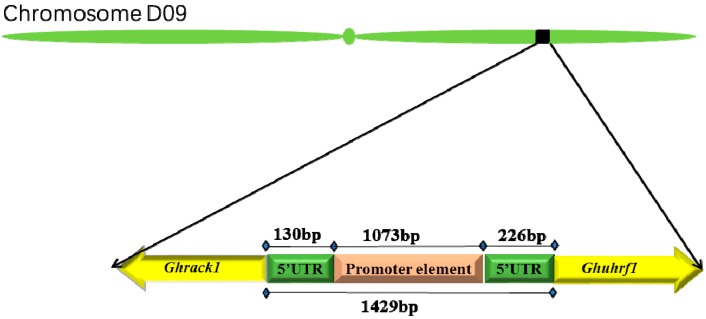
Schematic representation of the organization of the *Ghrack1* and *Ghuhrf1* genes on chromosome D09 of *Gossypium hirsutum*. The 1429 bp of the intergenic region and the core 1073 bp promoter are marked. In total, 226 bp and 130 bp 5′-UTRs were upstream of *Ghuhrf1* and *Ghrack1*, respectively.

Analysis of the genomic organization of the *Ghrack1* gene in the phytozome database indicated that a *Ghuhrf1* gene was located immediately upstream. The two genes were head-to-head on chromosome D09 of the *Gossypium hirsutum* genome. In silico analysis revealed that the translation initiation sites of these two adjacent genes were 1429 bp apart (Figure S1). Precise identification of transcription start sites (TSS) in *Ghrack1* and *Ghuhrf1* was through 5′-rapid amplification of cDNA ends (5′-RACE) (Figure S4). *Ghrack1* had a 130-bp 5′-UTR, and the *Ghuhrf1* gene contained a 5‘-untranslated region (5′-UTR) of 226 bp. The distance between the transcription start sites of the two genes was 1073 bp (Figure 1).

### 2.2. Relative Expression (Transcript) of the Ghrack1 and Ghuhrf1 Genes in Various Upland Cotton Tissues

The relative transcript abundance of these two (*Ghrack1* and *Ghuhrf1*) adjacent genes in the root, leaf, anther, stigma, and fiber tissues from different periods of the upland cotton K312 was assayed by semiquantitative PCR (Polymerase Chain Reaction) and quantitative real-time PCR (qRT-PCR) to evaluate their possible functional roles during development and growth.

**Figure 2:**
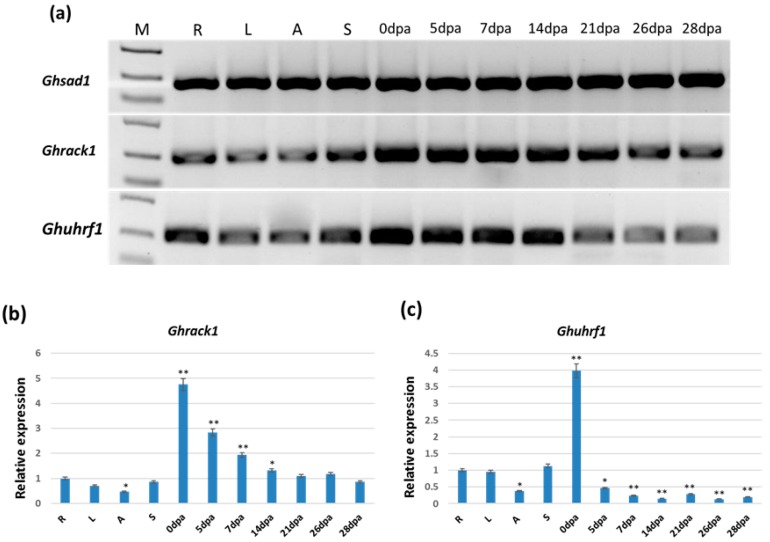
Semiquantitative PCR and qRT-PCR (quantitative real-time PCR) data of the transcript levels of the *Ghrack1* and *Ghuhrf1* genes in different tissues of *Gossypium hirsutum*. (**a**) Semiquantitative PCR survey of various cotton tissues for the detection of *Ghrack1* and *Ghuhrf1* transcripts. *Ghsad1* was used as a control. (**b**,**c**) Relative transcript abundance of two *Gossypium hirsutum* genes, that is, *Ghrack1* (**a**) and *Ghuhrf1* (**c**), detected in various cotton tissues by qRT-PCR. The data represent the relative expression of the *Ghrack1* and *Ghuhrf1* transcripts ± SD of three biological replicates in each tissue (*n* = 3). Asterisks and double asterisks indicate significant deviations from the root at *p* < 0.05 and *p* < 0.01, respectively, using the Student’s *t*-test for comparisons between the root and other tissues separately for both genes. R (root), L (leaf), A (anther), S (stigma), 0 dpa (0 dpa fiber), 5 dpa (5 dpa fiber), 7 dpa (7 dpa fiber), 14 dpa (14 dpa fiber), 21 dpa (21 dpa fiber), 26 dpa (26 dpa fiber), and 28 dpa (28 dpa fiber).

The semiquantitative PCR results showed that the *Ghrack1* gene had a higher expression level in the fiber tissues of various periods, but a lower expression level in other tissues. The transcript abundance of *Ghuhrf1* was highest in the initiation differentiation and elongation stage of fiber. For the semiquantitative PCR analysis, the *Ghsad1* gene was used as an internal control, and its expression level was found to be stable in all tissues (Figure 2a).

The qRT-PCR results showed that the high expression level of the *Ghrack1* gene was exhibited in the initiation-differentiation stage of fiber (Figure 2b). Compared with the relative expression of *Ghrack1* in the root, the relative transcript abundance was significantly higher (*p* < 0.01) in the 0 dpa (days post-anthesis), 5 dpa, and 7 dpa fibers and moderately higher (*p* < 0.05) in the 14 dpa fiber. This result was consistent with the semiquantitative PCR results. The *Ghuhrf1* transcript abundance was highest in the 0 dpa fiber, but there was a lower expression level in other periods of fiber and tissues (Figure 2c). Compared with the relative expression of *Ghuhrf1* in the root, the relative transcript abundance was significantly lower (*p* < 0.01) in the 7–28 dpa fibers and moderately lower (*p* < 0.05) in the anther and 5 dpa fiber, but significantly higher in the 0 dpa fiber (*p* < 0.01). These results somewhat differed from the semiquantitative PCR results, potentially due to the lower sensitivity of semiquantitative PCR. Thus, because of they shared an identical DNA region, the genes in both orientations which were driven by the same promoter exhibited similar expression profiles.

### 2.3. Sequence Analysis of the Cloned Promoter GhZU

**Figure 3:**
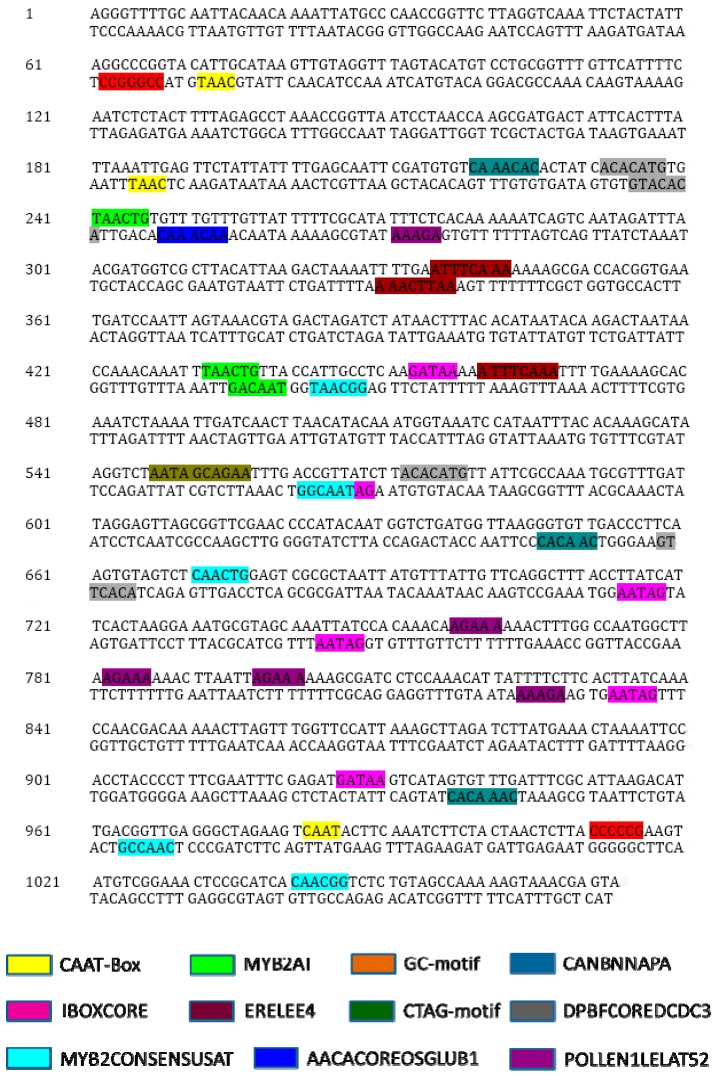
Sequence analysis of the cloned promoter GhZU. The putative cis-acting elements in both orientations of the promoter GhZU determined by the softwares PLACE and PlantCARE.

The intergenic region between *Ghrack1* and *Ghuhrf1* was cloned from *Gossypium hirsutum* K312 and named GhZU. The DNA sequencing analysis showed the high G+C content (38.7%) and three CpG islands in GhZU. The GhZU promoter fragment was submitted to PLACE (https://sogo.dna.affrc.go.jp/cgi-bin/sogo.cgi?lang=en&pj=640&action=page&page=newplace) and PlantCARE (http://bioinformatics.psb.ugent.be/webtools/plantcare/html/) to predict putative cis-elements involved in the regulation of gene expression (Figure 3). Potential regulatory elements were identified within GhZU (Table S2), including core elements, such as the CAAT-Box and GC-Box, but no TATA-Box. Some cis-elements were known to be involved in the growth and development of cotton fiber, such as MYB2AT, L1BOXATPDF1, and MYB2CONSENSUSAT. In addition, many elements in GhZU had been shown to participate in tissue-specific expression, such as ROOTMOTIFTAPOX1 (organ-specific gene expression), POLLEN1LELAT52 (anther-specific gene expression), and AACACOREOSGLUB1 and CANBNNAPA (the expression of endosperm-specific genes). A few elements were related to the ethylene response induced by cis-regulatory elements (ERELEE4). The predicted results of the 5’UTRs of *Ghrack1* and *Ghuhrf1* are presented in the supplementary materials (Figure S2). We defined the transcriptional orientation of the *Ghuhrf1* gene as forward and named it GhZUf, and the transcriptional orientation of *Ghrack1* as reverse and named it GhZUr.

### 2.4. GhZU Drives the Transient Expression of the Reporter Genes in Both Orientations

**Figure 4:**
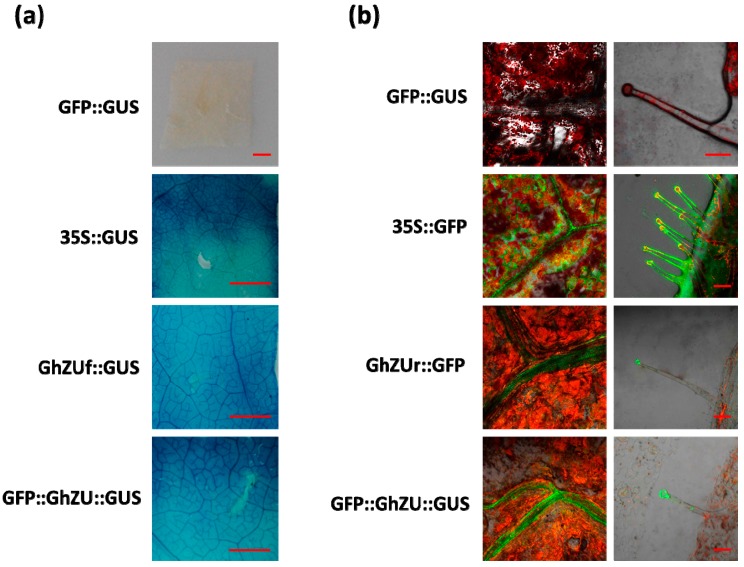
Transient expression of the *gus* gene and *gfp* gene in tobacco leaves, using the epidermis infection method. (**a**) Schematic diagram of the promoter-reporter gene constructs GFP::GUS, 35S::GUS, GhZUf::GUS, and GFP::GhZU::GUS, used for the transient assay in the *N. benthamiana* leaf using the pCambia1305 vector. (**b**) Schematic diagram of the promoter-reporter gene constructs GFP::GUS, 35S::GFP, GhZUr::GFP, and GFP::GhZU::GUS, used for the transient assay in the *N. benthamiana* leaf using the pCambia1305 vector. Below each construct, a representative assay of transient *gus* expression detected histochemically, transient *gfp* expression detected based on fluorescence imaging via the *Agrobacterium* infiltration assay in *N. benthamiana* leaf, and the respective promoter, no promoter, 35S promoter, *Ghuhrf1* promoter (GhZUf), and *Ghrack1* promoter (GhZUr) activities are shown.

*Agrobacterium* strains carrying the transient expression vector were introduced into young leaves of tobacco plantlets (6–8 leaf stage) by the infiltration transient expression assays. Histochemical GUS assays and GFP detection were performed 3 days after infection. The histochemical GUS assay results showed an obvious blue color in the whole leaf that was infected with *Agrobacterium* containing the vectors GhZUf::GUS and GUS::GhZU::GFP (Figure 4a). The GFP fluorescence detection results revealed green fluorescence in the leaves infected by *Agrobacterium* containing the vectors GhZUr::GFP and GUS::GhZU::GFP, whereas the *gfp* gene driven by GhZUr was preferentially expressed in leaf trichomes and veins, and no fluorescence signal was detected in other leaf sites (Figure 4b and Figure S3).

No detectable histochemical GUS staining was visualized in infected leaves from the untransformed control tobacco plants; however, strong histochemical GUS staining under CaMV35S was observed (Figure 4a). No detectable GFP fluorescence was visualized in infected leaves from the untransformed control tobacco plants, but strong GFP fluorescence under CaMV35S was observed (Figure 4b and Figure S3).

### 2.5. Detection and the Copy Number Analysis of Transgenic Arabidopsis thaliana

In order to obtain transgenic positive plants, we used the *gus* gene as the target gene and designed primers to identify them by PCR amplification. Finally, 246 transgenic positive plants were screened out from 320 individuals (Figure S4).

**Figure 5:**
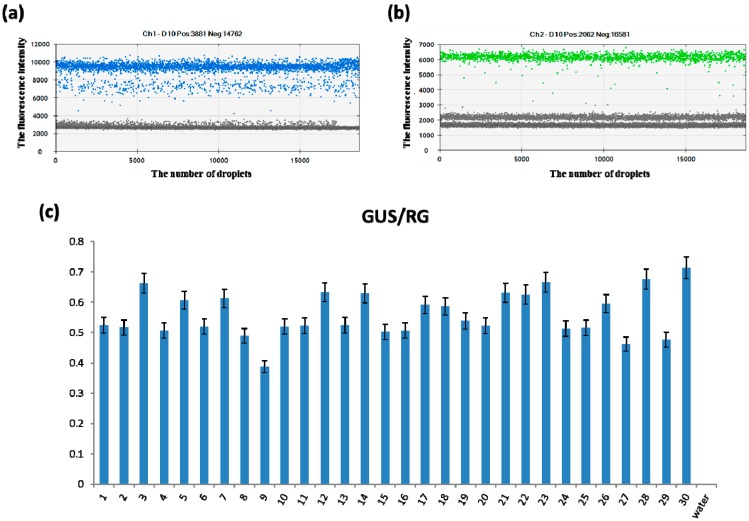
Detection of the copy number of transgenic *Arabidopsis thaliana* using the droplet digital PCR method. (**a**) Internal reference gene; (**b**) *gus* gene; (**c**) thirty selected single-copy *gus* gene individuals. The gray signal in the map represents the micro-droplets that had not been amplified by PCR, and the system was considered a negative signal; the blue signal shows the FAM fluorescence signal, and the green signal shows the HEX fluorescence signal representing the PCR amplification. The system was considered a positive signal.

According to the *Arabidopsis* single-copy gene *RG* (*AT1G03400.1*) and the target gene *gus*, we designed a primer probe for a droplet digital PCR. The amplification results showed that the designed primer probe had high specificity, and the system could clearly distinguish between positive and negative microdrops (Figure 5a,b). The number of microdrops generated in the experiment exceeded 13,000, thus meeting the requirements of the Poisson distribution, and the relative standard deviation (RSD) value of the number of droplets formed by three replicates was less than 0.25, meeting the requirements of EU (European Union) nucleic acid molecular testing. The above results show that the droplet digital PCR system was stable and repeatable, and that the data was reliable [21,22,23,24].

*Arabidopsis thaliana* is a diploid plant, and the RG base exists in the form of a single copy. In the T0 generation of the transgenic *Arabidopsis*, the target gene *gus* is hemizygous. Theoretically, the content of the gene in the genome should be 1/2 RG to demonstrate that the transferred *gus* gene is a single-copy gene. Subsequently, pure and single-copy *gus* transgenic *Arabidopsis* plants were obtained through selfing. Using an established droplet digital PCR system to calculate the copy number of the target gene, 162 single-copy *gus* individuals were selected from 246 positive seedlings, and 30 individuals were selected for the subsequent experiments (Figure 5c).

### 2.6. Function Analysis of the Bidirectional Promoter GhZU in Transgenic Arabidopsis thaliana

**Figure 6:**
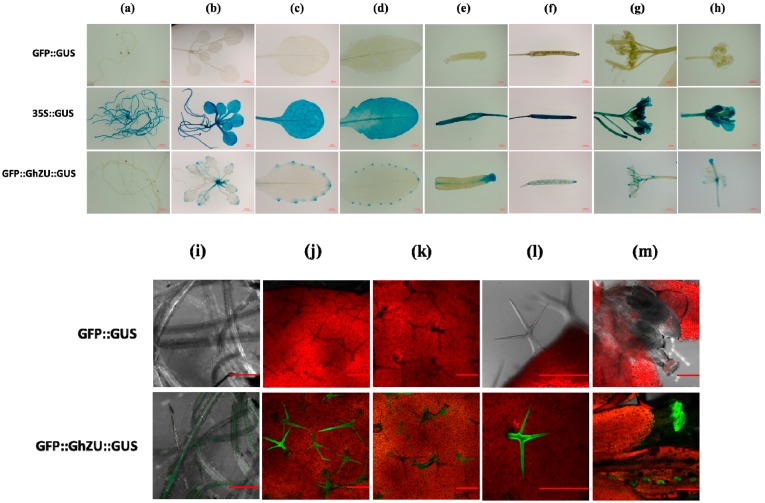
Localization of GUS and GFP in vegetative and reproductive tissues of GFP::GUS, 35S::GUS, and GFP::GhZU::GUS transgenic *Arabidopsis* plants. (**a**–**h**) Histochemical GUS localization data of GFP::GUS (up), 35S::GUS (middle), and GFP::GhZU::GUS (down) in vegetative and reproductive tissues of *Arabidopsis* plants. (**a**) Root region; (**b**) two-week-old *Arabidopsis* seedlings; (**c**) young leaf; (**d**) old leaf; (**e**) developing silique; (**f**) mature silique; (**g**) inflorescence; and (**h**) flower. Histochemical GUS images of GFP::GUS (up), 35S::GUS (middle), and GFP::GhZU::GUS (down) are shown. (**i**–**m**) Confocal laser-scanning microscopic analysis of *gfp* expression under GFP::GUS and GFP::GhZU::GUS in *Arabidopsis* plants. (**i**) Root, (**j**) young leaf trichomes, (**k**) old leaf trichomes, (**l**) trichomes, and (**m**) flower. Green fluorescence images of GFP::GUS (up) and GFP::GhZU::GUS (down) are shown.

To evaluate the tissue-specific nature of the bidirectional promoter, independent transgenic *Arabidopsis* lines were generated for the constructs 1305.1-GFP::GUS, 1305.1-35S::GUS, and 1305.1-GFP::GhZU::GUS. In the GFP::GhZU::GUS plants, during the growth and development of *Arabidopsis*, strong histochemical GUS staining under GhZUf was observed in the leaf tip, apical meristem, stigma, and petiole base regions (Figure 6a–h). In contrast, strong GFP expression of GhZUr was detected in the young leaf trichomes and old leaf trichomes, and weak GFP expression was detected in the whole roots and lateral roots (Figure 6i–m, Figure S3). In the reproductive tissues of *Arabidopsis*, GFP expression under GhZUr showed weak expression in the anther, stigma, and developing silique, whereas GUS expression under GhZUf was detected in the floral bud, stigma, and developing silique (Figure 6h). 

No detectable histochemical GUS staining was visualized in various tissues from the untransformed control *Arabidopsis* plants, including the root, leaf, flower, and immature and mature silique. However, strong histochemical GUS staining under CaMV35S was observed in all tissues during *Arabidopsis* growth and development (Figure 6a–h). Also, no detectable GFP fluorescence was observed in the vegetative tissues from the untransformed control *Arabidopsis* plants (Figure 6i–m, Figure S3).

The tissue-specific expression pattern of the bidirectional promoter GhZU was confirmed with the reporter genes (*gus* and *gfp*). According to the expression of the *gus* gene driven by GhZUr, GUS was mainly expressed in sites of vigorous plant growth, such as the leaf tip, apical meristem, and petiole base regions. GFP was predominantly expressed in young and vigorously growing plant tissues, such as young leaf trichomes. Therefore, the intergenic region GhZU was indeed found to be a bidirectional promoter, and was able to drive the reporter genes’ expression and in a similar manner according to its orientation.

### 2.7. Relative Expression Level of the gfp and gus Genes in Various Arabidopsis Tissues

The transcript abundance of the reporter genes *gfp* and *gus* were measured to better understand the regulation of the expression of these two tissue-specific genes (*Ghrack1* and *Ghuhrf1*) under the bidirectional promoter GhZU. The relative transcript abundance of these two reporter genes (*gfp* and *gus*) in various tissues (root, young leaf, older leaf, flower, developing silique, and mature silique) were assayed by quantitative real-time PCR (qRT-PCR), using gene-specific primers to evaluate the tissue-specific expression of the bidirectional promoter in both orientations.

**Figure 7:**
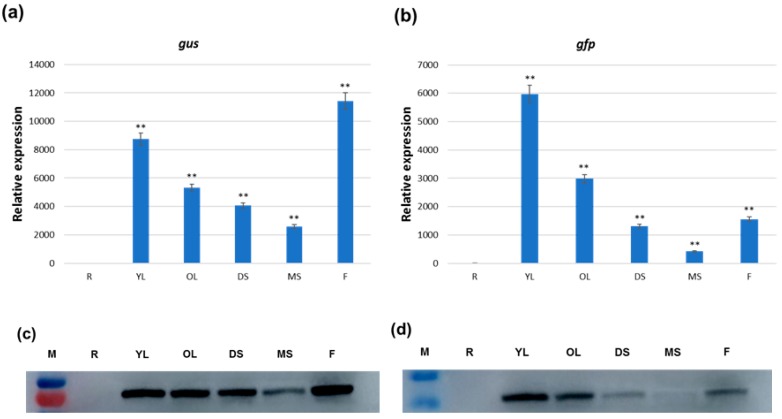
GUS and GFP expression in various tissues of transgenic *Arabidopsis* plants generated for the constructs GFP::GhZU::GUS. (**a**,**b**) Relative expression (transcript) of two reporter genes, that is, *gus* (**a**) and *gfp* (**b**), detected in various *Arabidopsis* tissues by qRT-PCR. The data represent the relative expression of the *gus* and *gfp* transcripts ± SD of three biological replicates of each tissue (*n* = 3). Asterisks and double asterisks indicate significant deviations from the root at *p* < 0.05 and *p* < 0.01, respectively, using Student’s *t*-test for comparisons between the root and other tissues separately for both genes. (**c**,**d**) Relative expression (protein) of two reporter genes, that is, *gus* (**c**) and *gfp* (**d**), detected in various *Arabidopsis* tissues by protein hybridization. R (root), YL (young leaf), OL (old leaf), DS (developing silique), MS (mature silique), and F (inflorescence).

The expression level of the *gus* gene was in the following order: maximum expression in the flower tissue, followed by that of the young leaf, older leaf, developing silique, and mature silique; however, there was almost no expression in the root tissue (Figure 7a). The *gfp* transcript abundance was highest in the young leaf, followed by the older leaf, developing silique, flower tissue, and mature silique, whereas the lowest expression was detected in the root tissues (Figure 7b). These data indicate that this bidirectional promoter directed gene expression in an orientation-dependent manner during the *Arabidopsis’* development and growth.

### 2.8. Detection of the Expression of the gus and gfp Genes in Various Tissues of Arabidopsis thaliana by Western Blot

The relative protein abundance of these two reporter genes in various tissues (root, young leaf, older leaf, flower, developing silique, and mature silique) were assayed by western blot using protein-specific antibodies to evaluate the tissue-specific expression of the bidirectional promoter in both orientations.

The western blot analysis using an anti-GUS antibody revealed strong GUS expression in the young leaves, older leaves, developing silique, and floral tissues, and moderate expression in the mature silique. However, no detectable GUS protein was visualized in the root (Figure 7c). Using an anti-GFP antibody revealed that GFP expression was highest in the young leaves, and had moderate expression in the old leaves. Whereas the lowest expression was detected in the developing silique and floral tissues, and GFP protein expression was not detected in the root (Figure 7d). The western blot results differed from the results obtained in the fluorescence quantitative analysis, potentially because the total protein from the plants degraded during extraction, or the protein expression was too weak and the sensitivity was too low, making it impossible to detect.

## 3. Discussion

With the rapid development of genome-sequencing technology today, more and more species have been completed genome sequencing. Functional studies of intergenic regions have attracted the most attention of researchers [11], and the role of noncoding DNA in phenotypic evolution has been reported many times in the literature [25]. The orientation of flanking genes may influence the evolution of intergenic regions, in which cis-regulatory elements are likely to be located [26,27]. The head-to-head clustering of genes in which two adjacent genes are separated by a short intergenic distance have been determined by the direction of gene expression in different transcription configurations, and are also prevalent and conserved in many eukaryotes, including yeasts, plants, invertebrates, and vertebrates [28]. With the availability of the complete genome sequences of several organisms, the functionality of intergenic regions has attracted increasing attention. Compared with unidirectional promoters, bidirectional promoters have some specific structural characteristics. Generally, the length of a bidirectional promoter is largely within 1000 bp in humans, but often longer than 1000 bp in plants [5,10]. The bidirectional promoter that drives rice chymotrypsin protease inhibitor genes (*OCPI1* and *OCPI2*) is 1126 bp [29]. Also, an intergenic region (1258 bp) shared by At4g35985 and At4g35987 in *Arabidopsis thaliana* is a tissue-specific and stress-inducible bidirectional promoter analyzed in transgenic *Arabidopsis* and tobacco plants [11]. High C+G content is also an important feature of bidirectional promoters. Yang et al. found that 70.8% of human bidirectional promoters have a G+C content exceeding 60%, and that CpG-islands are present in 90% of bidirectional promoters [5,30]. The C+G content in bidirectional promoters in rice, *Arabidopsis*, and *Poplar* are 55%, 37%, and 48%, respectively, which are significantly higher than its content in randomly selected promoters [15]. The G+C content of bidirectional promoters are 55%, 48.2%, 31%, and 34% in sorghum, rice, soybean, and *Arabidopsis* genomes, respectively, while those in randomly selected promoters are 46.5%, 44.3%, 29.8%, and 32.1%, respectively. The G+C content of bidirectional promoters are generally higher than those in randomly selected promoters. This trend is consistent with that of bidirectional promoters in maize [18]. There is a growing interest in function analysis of intergenic regions, which would not only help us get a better understanding of divergent transcription, but also help us turn the information into a new tool for the manipulation of genomes [31]. In this study, the region shared by two adjacent genes *Ghrack1* and *Ghuhrf1* on chromosome D09 of *Gossypium hirsutum* was found to function as a promoter in both orientations. The intergenic region GhZU between the TSS of *Ghrack1* and *Ghuhrf1* was found to have a 38.7% C+G content, three CpG islands, and no TATA-box motif, which is why we conducted a critical analysis of this intergenic sequence.

We cloned the 1429 bp intergenic region sequence between the ATGs of the head-to-head gene pair. The intergenic region sequence was submitted to PLACE and PlantCARE to predict the putative cis-elements involved in the regulation of gene expression. Potential regulatory elements were identified within GhZU, including core elements such as the CAAT-Box and GC-Box, but no TATA-Box. Some cis-elements have been known to be involved in the growth and development of cotton fiber, such as MYB2AT, L1BOXATPDF1, and MYB2CONSENSUSAT; therefore, we linked the intergenic region sequence to the reporter genes in both orientations. We transformed the expression vectors into tobacco and *Arabidopsis thaliana*, where for tobacco, we demonstrated that the intergenic sequences had bidirectional promoter activity, and for transgenic *Arabidopsis thaliana*, the *gus* gene was mainly expressed in regions and tissues with more vigorous growth, and weak or no expression was observed in the mature tissues. Consistent with the *Ghuhrf1* gene expression in cotton, this gene was mainly expressed during the initial stage of fiber progenitor cells, and the expression gradually decreased over time. Therefore, based on the expression of the *gus* gene in *Arabidopsis thaliana* and the *Ghuhrf1* gene in cotton, the GhZUf promoter was shown to be a tissue- and period-specific promoter, whereas the *gfp* gene was mainly expressed in the epidermal hairs at the beginning of growth and the newly developed silique, but was not expressed in the mature tissues. Consistent with the expression of the *Ghrack1* gene in cotton, this gene was mainly expressed during the early stage of fiber development, followed by a gradual decline with the growth and development of the fiber until the fiber matured. Many researchers have found that the development mechanism of *Arabidopsis* epidermis hair is similar to that of cotton fiber [32]. Therefore, based on the *gfp* gene expression in *Arabidopsis thaliana* and the *Ghrack1* gene expression in cotton, the GhZUr promoter was determined to be a dominant fiber-expression promoter. In summary, the GhZU promoter is an orientation-dependent bidirectional promoter in cotton and *Arabidopsis,* and has certain dependencies in both directions of initiation; however, some differences exist in time and space. The expression patterns of GhZUf and GhZUr should be confirmed in future studies through its stable expression in transgenic cotton.

To improve plants by using molecular biology and genetic engineering methods, transferring multiple genes concomitantly is often necessary. Gene fusion technology or gene stacking technology is commonly used to recombine or superimpose two or more functional genes or to use multiple transgenes to transfer multiple functional genes into the same plant, which is usually completed by using a high-expression efficiency promoter [33]. Previous studies have shown that the presence of a homologous sequence between two promoters in the plant may cause “co-inhibition” of gene expression, leading to partial gene silencing [4]. Compared with unidirectional promoters, bidirectional promoters improve the efficiency of biotechnological improvements by regulating many genes, which is why bidirectional promoters have attracted considerable attention. The bidirectional promoters analyzed in this study received attention due to their similar tissue-specific expression patterns and variation in the expressional magnitude in both orientations. In one direction (GhZUf), the promoter is very active in the tip of the leaf and apical meristem regions, while in the other direction (GhZUr), the promoter is strongly expressed in young tissues, such as young leaf epidermal hairs. To increase the quantity and quality of fibers, several genes relatived with fiber growth and development can be simultaneously expressed in two directions to obtain more high-quality fibers. Therefore, the bidirectional promoters shared regulatory elements of expression in the opposite direction, and the genes driven by bidirectional promoters could be better expressed in the host cell under selective pressure than by other synthetic promoters. The bidirectional promoters could become the preferred promoters for transgene breeding in the future through the use of multigene co-transformation, which is invaluable in the field of genetic engineering research and its applications.

## 4. Materials and Methods 

### 4.1. Plant Materials and Growth Conditions 

*Nicotiana benthamiana* was kindly provided by Professor Luo’s laboratory from the College of Life Science of Southwest University. Tobacco and cotton were grown in a greenhouse under a 16 h light/8 h dark cycle at 28–30 °C. Wild-type *Arabidopsis thaliana* (Col-0) and seeds of transgenic plants were surface-sterilized with 75% (*v*/*v*) ethanol for 8 min, followed by 1 min with 95% (*v*/*v*) ethanol. The sterilized *Arabidopsis* seeds were plated on containing 1/2 Murashige and Skoog medium. Seeds were stratified at 4 °C for 2 days, and the plates were subsequently transferred to a plant growth incubator under a 16 h light/8 h dark cycle at 23 °C. 

### 4.2. Transcript Analysis by Semiquantitative PCR and Quantitative Real-Time PCR (qRT-PCR) in Upland Cotton

The total RNA was extracted from the root, leaf, anther, stigma, and fiber tissues at different periods (0, 5, 7, 14, 21, 26, and 28 dpa) from upland cotton K312, and cDNA was generated using a primescript^TM^ RT reagent kit and gDNA eraser (TaKaRa, Dalian, China) following the manufacturer’s instructions. For quantitative measurements of the *Ghrack1*-specific transcript, forward (q-Ghrack1-F) and reverse (q-Ghrack1-R) primers were used. For the quantitative measurements of the *Ghuhrf1*-specific transcript, forward (q-Ghuhrf1-F) and reverse (q-Ghuhrf1-R) primers were also used. *Sad1* was used as a control with the primers q-sad-F and q-sad-R [34]. Quantification of the *Ghrack1* and *Ghuhrf1* transcript levels by semiquantitative PCR was also performed. Each reaction was performed in triplicate on a T-100 thermal cycler (BIO-RAD, CA, USA) using the following conditions: denaturation at 95 °C for 5 min, followed by 30 cycles of denaturation at 95 °C for 30 s, annealing at 60 °C for 30 s, and extension at 72 °C for 30 s. The relative transcript abundance was assessed by semiquantitative PCR, following a published protocol [35]. PCR was performed in four replicates and repeated in three biological samples. Quantitative real-time transcription PCR (qRT-PCR) was carried out to confirm the sequencing data identifying the DEGs. Using the first cDNA strand as a template, qRT-PCR was performed on an ABI7500 Real-Time System (Applied Biosystems, USA) using a SYBR premix Ex Taq kit (TaKaRa, Dalian, China). For the qRT-PCR analysis, the *sad1* gene was used as an internal control, and the relative quantification method was used to assess the fold changes in the target genes. Three technical replicates were performed using one biological sample. PCR was performed for 30 s at 95 °C, followed by 40 cycles of 95 °C for 5 s and 60 °C for 34 s. All primers are listed in Table S1.

### 4.3 Precise Identification of Transcription Start Sites in Ghrack1 and Ghuhrf1 by 5′-Rapid Amplification of cDNA Ends (5'-RACE)

Total RNA was extracted from the leaves of upland cotton K312 using an RNAprep pure plant kit (TIANGEN, Beijing, China), following the manufacturer’s instructions. Total RNA was treated with calf intestinalphosphatase (CIP) to remove the 5′ phosphate from partial transcripts. Dephosphory RNA was treated with tobacco acid pyrophosphatase (TAP), which removed the 5′ cap from capped mRNA and exposed the 5′ phosphate. The GeneRacer™ RNA Oligo (Thermo Fisher Scientific, MA, USA) was ligated to the TAP-treated mRNA with T4 RNA ligase. A cDNA template was generated by reverse transcription using SuperScript™ II RT and either the GeneRacer™ Oligo dT Primer or the gene-specific primer (GSP1 and GSP2). 5′ ends were PCR-amplified from these cDNA templates with a primer for the GeneRacer™ RNA Oligo (GeneRacer™ 5′ Primer) and gene-specific primer. Only cDNA containing the GeneRacer™ RNA Oligo sequence were amplified [36,37].

### 4.4. Cloning and Sequence Analysis of the Intergenic Region between Ghrack1 and Ghuhrf1 

The primer pairs GhZU-F and GhZU-R were designed according to the DNA sequence between the *rack1* and *uhrf1* genes in the *Gossypium hirsutum* genome. The intergenic region between *Ghrack1* and *Ghuhrf1* was amplified using genome DNA from *Gossypium hirsutum* K312 as a template and GhZU-F/GhZU-R as primers. The PCR amplification with Phanta Max Super-Fidelity DNA Polymerase was performed according to the manufacturer’s instructions (Vazyme, Nanjing, China), under the following reaction conditions: 5 min at 95 °C, followed by 30 cycles of 30 s at 95 °C, 30 s at 58 °C, 2 min at 72 °C, and 10 min at 72 °C. The amplified DNA fragment was sequenced and analyzed using the software DNAman, PlantCARE [38], PLACE [39], and CpGPlot/CpGReport/Isochore.

### 4.5. Construction of Plant Expression Vectors

The target DNA fragments of the forward orientation promoter GhZUf and reverse orientation promoter GhZUr were cloned using the corresponding primers and template shown in Table S1. GhZUr-*gfp* was amplified by nested PCR, using previous PCR products of GhZUr and *gfp* as templates and GhZUr-F/gfp-R as primers. All target DNA fragments were ligated into the cloning vector pMD18-T (TaKaRa, Dalian, China) and sequenced.

The target DNA fragments of GhZUf, GhZUr, *gus,* and *gfp* were inserted between the *Xba*I, *Nco*I, and *Sph*I sites of the plant expression vector pCambia1305.1 (Figure S5) using a homologous recombinant enzyme (TaKaRa, Dalian, China) to form the vector GhZUf::gus, GhZUr::gfp, gfp:GhZU::gus, and gfp::gus, respectively.

### 4.6. Transient Expression in Tobacco and Stable Expression in Arabidopsis

The final plant expression vectors were mobilized into the *Agrobacterium tumefaciens* strain GV3101 by using the freeze-thaw method. First, we transformed the vector into tobacco for the transient expression experiments. The transformed bacteria were grown on YEB medium containing 50 mg/L kanamycin, 100 mg/L rifampicin at 28 °C, and 220 rpm overnight. The cultures were diluted 1:100 with YEB and allowed to grow to an absorbance (measured at 600 nm) of <0.8. The young leaf epidermis nearest to the top of *Nicotiana benthamiana* at approximately the 6–8 leaf stage was used for infection with *A. tumefaciens*. The infection was stopped when at least two-thirds of the target leaf was consumed by *A. tumefaciens*. After the infection, the plants were cultured at a high humidity for 12 to 16 h in the dark, and then cultured under normal conditions for 3 days. Then, we transformed the vector into *Arabidopsis* for the stable expression experiments using a standard floral-dipping method [40]. The transformants were selected on MS medium supplemented with 50 mg/L kanamycin. Transgenesis was confirmed via PCR using GhZU-F/R. Transgene homozygotes selected from the T3 generation were used for the analysis of *gfp* and *gus* expression.

### 4.7. Detection and Copy Number Analysis of Transgenic Arabidopsis-Positive Plants

The gDNA was extracted from different plants using a DNAsecure plant kit (TIANGEN, Beijing), following the manufacturer’s instructions. These gDNA were used as templates and GUS-JC-F/GUS-JC-R as primers. The PCR amplification with Phanta Max Super-Fidelity DNA Polymerase was performed according to the manufacturer’s instructions (Vazyme, Nanjing, China) under the following reaction conditions: 5 min at 95 °C, followed by 30 cycles of 30 s at 95 °C, 30 s at 58 °C, 1 min at 72 °C, and 5 min at 72 °C.

The single-copy gene RG (AT1G03400) of *Arabidopsis thaliana* was selected as the internal reference gene, while the corresponding primers and probes were designated as RG-F/R, and the RG-probe fluorescent markers were designated as FAM (6-carboxy-fluorescein). The target gene probes and primers, including the GUS-F/R, GUS-probe, and fluorescently labeled HEX (hexachloro fluorescein), were designed using primer premier 5.0 software. A 20-μL droplet digital PCR probe reaction system was prepared, mixed well, and added to a microdroplet generator. Then, a 70-μL droplet of oil was added to the corresponding wells, which were covered with special pads and placed in a droplet generator. The oil droplets were transferred to a 96-well plate and placed with the red-labeled side of the membrane facing up, which was fixed and sealed with a heat sealer. The procedure consisted of an incubation at 180 °C for 10 s. Each reaction was performed in triplicate on a QX200 (BIO-RAD). The rate at which the PCR device raised and lowered the temperature was controlled within the range of ≤2.5 °C/s. The reaction conditions consisted of predenaturation at 94 °C for 10 min, denaturation at 94°C for 30 s, annealing at 62 °C for 60 s for 40 cycles, and incubation at 98 °C for 10 min. Then, the microdrop reader read the droplet signal and analyzed the experimental results using QuantaSoft software [41,42].

### 4.8. Histochemical GUS Assay and GFP Detection

The GUS assay was performed as described by Jefferson et al. [43]. Plant tissues or leaf sections were soaked in X-gluc solution, sealed closely to avoid evaporation, incubated overnight at 37 °C in the dark, and fixed in a formalin-isopropyl alcohol-glacial acetic acid (FAA) solution for 15 min. To remove chlorophyll, the fixed leaf sections were rinsed successively in 75%, 85%, 95%, and 100% ethanol.

For GFP detection, samples from various stages of plant development were placed on a slide glass and covered with a coverslip [11]. The laser-scanning confocal microscope Axio LSM 700 (Zeiss Co., Ltd., Jena, Germany) was used for the observations.

### 4.9. RNA Isolation and Quantitative Real-Time PCR (qRT-PCR) in Various Transgenic Arabidopsis Tissues

The total RNA was extracted from different plant tissues using an RNAprep pure plant kit (TIANGEN, Beijing, China) following the manufacturer’s instructions. For the quantitative measurements of the *gfp* transcript, the primer pair of q-gfp-F/R were used. For the quantitative measurements of the *gus* transcript using q-gus-F/R, the relative transcript abundance was assessed by qRT-PCR following a published protocol [35]. PCR was performed in four replicates and repeated in three biological samples. The transcript levels were measured following the comparative Ct method (Applied Biosystems bulletin, MA, USA). To normalize the amount of total RNA in all *Arabidopsis* samples, the *actin* gene-specific forward primer q-actin-F and reverse primer q-actin-R were used [44].

### 4.10. SDS-Polyacrylamide Gel Electrophoresis and Immunoblot Analysis

SDS-polyacrylamide gel electrophoresis was performed using 10% polyacrylamide gels, as previously described [45]. 40 micrograms of protein from young leaf, older leaf, flower, root, developing silique, and mature silique tissues from transgenic *Arabidopsis* plants were subjected to 10% SDS-polyacrylamide gel electrophoresis for the western blot. The Rubisco large subunit (LSU) was stained with Ponceau S as an internal control for loading uniformity.

For the determination of GFP expression in different tissues, a western blot analysis was performed using an anti-GFP tag mouse monoclonal antibody (Plant Specific) (1:3000–5000) from Beijing Emarbio Science & Technology Co., Ltd., as well as a horseradish peroxidase-conjugated goat anti-rabbit secondary antibody (1:5000–10,000), and detected using a chemiluminescent reagent (abm, Vancouver, BC, Canada) following a published protocol [46].

For the determination of GUS expression in different tissues, a western blot analysis was conducted using an anti-GUS tag rabbit polyclonal antibody from Agrisera antibodies (Agrisera, Vannas, Swedish) (1:10,000) and a horseradish peroxidase-conjugated goat anti-rabbit secondary antibody (1:5000–10,000), and detected using a chemiluminescent reagent, following a published protocol.

## Supplementary Materials

Supplementary materials can be found at http://www.mdpi.com/1422-0067/19/11/3291/s1.
