# Supplementary material for: Structural and Functional Analysis of a Bidirectional Promoter from Gossypium hirsutum in Arabidopsis
Source: Int J Mol Sci. 2018 Oct 23;19(11):3291. doi: 10.3390/ijms19113291 (PMC6274729; doi:10.3390/ijms19113291)
Supplement: Supplementary file 1 [file ijms-19-03291-s001.zip › Supplementary materials/Figure S1.docx]

TTTCGTCGGCGCTTGCGGCGGGAGCGGCCTTTAGGGTTTTGAATGATAAAATGATAGGTCAGCGGAGAATGAAAAGCAAGAATGTAAGGCATAAGAAAGTATATATAGTACCGTTGGTATATTGGGAATTAGGGTTTTGCAATTACAACAAAATTATGCCCAACCGGTTCTTAGGTCAAATTCTACTATTAGGCCCGGTACATTGCATAAGTTGTAGGTTTAGTACATGTCCTGCGGTTTGTTCATTTTCAATCTCTACTTGTAGAGCCTAAACCGGTTAATCCTAACCAAGCGATGACTATTCACTTTATGCCATTGAGTTCTATTATCTTGAGCAATTCGATGTGTCAAACACACTATCACACATGTGTAACTGTGTTTGTTTGTTATGTTTCGCATATTTCTCACAAACCATCAGTCAATAGATTTAACGATGGTCGCTTACATTAAGACTAAGGCCTTGAATTTCAAAACCAGCGACCACGGTGAATGATCCAATTAGTAAACGTAGACTAGATCTATAACTTTACACATAATACAAGACTAATAACCAAACAAATTGCACTGTTACCATTGCCTCAAGATAAACCGTTCAACGTTTGAAAAGCACAAATCTAACATTGATCAACTTAACATACAAATGGTAGCTCCATACGTTACACAAAGCATAAGGTCTAATAGCAGAATTTGACCGTTATCTTACACATGTTATTCGCCAAATGCGTTTGATTAGGAGTTAGCGGTTCGAACCCCATACAATGGTCTGATGGTTAAGGGTGTTGACCCTTCAAGTGTAGTCTCAACTGGAGTCGCGCTAAGTATGTTTATTGTTCAGGCTTTACCTTATCATTCACTAAGGAAATGCGTAGCAAATTATCCACAAACAAGAAAAAACTTTGGCCAATGGCTTAAGAAAACACTTACGTAGAAACAAGCGATCCTCCAAACATTAGTTTCTTCACTTATCAAACCAACGACAAACACTTAGTTTGGTTCCATTAAAGCTTAGATCTTATGAAACTAAACGTCCACCTACCCCTTTCGAATTTCGAGATGATAAGTCATAGTGTTTGATTTCGCATTAAGACATTGACGGTTGAGGGCTAGAAGTCAATACTTCAAATCTTCTACTAACTCTTACCCCCGAAGTATGTCGGAAACTCCGCATCACAACGGTCTCTGTAGCCAAAAAGTAAACGAGTAACAAAATGGCATAAATGTAAAAAAATGAATTAAAAAGAATAATATCGTCAATTCATATTTATCCTCTTCAGGAATTCAAAAAGTCTCTCAAATTCCATACCGAAGCCCCCCCGCCTCTCTCTCTCTATATATCTTTTCTCCTCTCGCCCACTCTTCCCTCCAGTCTCCGTCCCATCAAAATTCAACGTCTGCTATCTTGCGCCTCAAGCTCATTGTTTGTTTGCCA

**Figure S1** The sequence of an intergenic region (1429 bp) shared by *Ghrack1* and *Ghuhrf1*. It contains bidirectional promoter sequence (1073 bp, red), the 5’UTR of *Ghrack1* gene (130 bp, blue) and the 5’UTR of *Ghuhrf1* gene (226 bp, green)
