# Supplementary material for: Structural and Functional Analysis of a Bidirectional Promoter from Gossypium hirsutum in Arabidopsis
Source: Int J Mol Sci. 2018 Oct 23;19(11):3291. doi: 10.3390/ijms19113291 (PMC6274729; doi:10.3390/ijms19113291)
Supplement: Supplementary file 1 [file ijms-19-03291-s001.zip › Supplementary materials/Figure S2.docx]

a: The predicted results of the 5’UTR of *Ghrack1*

TTTCGTCGGCGCTTGCGGCGGGAGCGGCCTTTAGGGTTTTGAATGATAAAATGATAGGTCAGCGGAGAATGAAAAGCAAGAATGTAAGGCATAAGAAAGTATATATAGTACCGTTGGTATATTGGGAATT

130 base pairs

(+) = Current Strand

(-) = Opposite Strand

1 TTTCGTCGGCGCTTGCGGCGGGAGCGGCCTTTAGGGTTTTGAATGATAAA

(-) CGACGOSAMY3 [S000205](https://sogo.dna.affrc.go.jp/cgi-bin/sogo.cgi?sid=&pj=640&action=newPlaceSite&site=S000205) 4 CGACG

(-) LTRECOREATCOR15 [S000153](https://sogo.dna.affrc.go.jp/cgi-bin/sogo.cgi?sid=&pj=640&action=newPlaceSite&site=S000153) 5 CCGAC

(-) DRECRTCOREAT [S000418](https://sogo.dna.affrc.go.jp/cgi-bin/sogo.cgi?sid=&pj=640&action=newPlaceSite&site=S000418) 5 RCCGAC

(-) CBFHV [S000497](https://sogo.dna.affrc.go.jp/cgi-bin/sogo.cgi?sid=&pj=640&action=newPlaceSite&site=S000497) 5 RYCGAC

(-) DOFCOREZM [S000265](https://sogo.dna.affrc.go.jp/cgi-bin/sogo.cgi?sid=&pj=640&action=newPlaceSite&site=S000265) 29 AAAG

(-) TAAAGSTKST1 [S000387](https://sogo.dna.affrc.go.jp/cgi-bin/sogo.cgi?sid=&pj=640&action=newPlaceSite&site=S000387) 29 TAAAG

(-) TELOBOXATEEF1AA1 [S000308](https://sogo.dna.affrc.go.jp/cgi-bin/sogo.cgi?sid=&pj=640&action=newPlaceSite&site=S000308) 31 AAACCCTAA

(-) UP2ATMSD [S000472](https://sogo.dna.affrc.go.jp/cgi-bin/sogo.cgi?sid=&pj=640&action=newPlaceSite&site=S000472) 32 AAACCCTA

(+) GATABOX [S000039](https://sogo.dna.affrc.go.jp/cgi-bin/sogo.cgi?sid=&pj=640&action=newPlaceSite&site=S000039) 45 GATA

(+) GT1CONSENSUS [S000198](https://sogo.dna.affrc.go.jp/cgi-bin/sogo.cgi?sid=&pj=640&action=newPlaceSite&site=S000198) 45 GRWAAW

(+) IBOXCORE [S000199](https://sogo.dna.affrc.go.jp/cgi-bin/sogo.cgi?sid=&pj=640&action=newPlaceSite&site=S000199) 45 GATAA

51 ATGATAGGTCAGCGGAGAATGAAAAGCAAGAATGTAAGGCATAAGAAAGT

(+) GATABOX [S000039](https://sogo.dna.affrc.go.jp/cgi-bin/sogo.cgi?sid=&pj=640&action=newPlaceSite&site=S000039) 53 GATA

(+) QELEMENTZMZM13 [S000254](https://sogo.dna.affrc.go.jp/cgi-bin/sogo.cgi?sid=&pj=640&action=newPlaceSite&site=S000254) 56 AGGTCA

(-) WBOXNTERF3 [S000457](https://sogo.dna.affrc.go.jp/cgi-bin/sogo.cgi?sid=&pj=640&action=newPlaceSite&site=S000457) 57 TGACY

(-) WBOXNTCHN48 [S000508](https://sogo.dna.affrc.go.jp/cgi-bin/sogo.cgi?sid=&pj=640&action=newPlaceSite&site=S000508) 57 CTGACY

(-) WRKY71OS [S000447](https://sogo.dna.affrc.go.jp/cgi-bin/sogo.cgi?sid=&pj=640&action=newPlaceSite&site=S000447) 58 TGAC

(-) INRNTPSADB [S000395](https://sogo.dna.affrc.go.jp/cgi-bin/sogo.cgi?sid=&pj=640&action=newPlaceSite&site=S000395) 66 YTCANTYY

(+) DOFCOREZM [S000265](https://sogo.dna.affrc.go.jp/cgi-bin/sogo.cgi?sid=&pj=640&action=newPlaceSite&site=S000265) 73 AAAG

(+) POLLEN1LELAT52 [S000245](https://sogo.dna.affrc.go.jp/cgi-bin/sogo.cgi?sid=&pj=640&action=newPlaceSite&site=S000245) 94 AGAAA

(+) DOFCOREZM [S000265](https://sogo.dna.affrc.go.jp/cgi-bin/sogo.cgi?sid=&pj=640&action=newPlaceSite&site=S000265) 96 AAAG

(-) CACTFTPPCA1 [S000449](https://sogo.dna.affrc.go.jp/cgi-bin/sogo.cgi?sid=&pj=640&action=newPlaceSite&site=S000449) 98 YACT

101 ATATATAGTACCGTTGGTATATTGGGAATT

(-) CACTFTPPCA1 [S000449](https://sogo.dna.affrc.go.jp/cgi-bin/sogo.cgi?sid=&pj=640&action=newPlaceSite&site=S000449) 107 YACT

(-) CURECORECR [S000493](https://sogo.dna.affrc.go.jp/cgi-bin/sogo.cgi?sid=&pj=640&action=newPlaceSite&site=S000493) 108 GTAC

(+) CURECORECR [S000493](https://sogo.dna.affrc.go.jp/cgi-bin/sogo.cgi?sid=&pj=640&action=newPlaceSite&site=S000493) 108 GTAC

(-) MYB2CONSENSUSAT [S000409](https://sogo.dna.affrc.go.jp/cgi-bin/sogo.cgi?sid=&pj=640&action=newPlaceSite&site=S000409) 111 YAACKG

(-) MYBCOREATCYCB1 [S000502](https://sogo.dna.affrc.go.jp/cgi-bin/sogo.cgi?sid=&pj=640&action=newPlaceSite&site=S000502) 111 AACGG

(+) MYBCORE [S000176](https://sogo.dna.affrc.go.jp/cgi-bin/sogo.cgi?sid=&pj=640&action=newPlaceSite&site=S000176) 111 CNGTTR

(+) ROOTMOTIFTAPOX1 [S000098](https://sogo.dna.affrc.go.jp/cgi-bin/sogo.cgi?sid=&pj=640&action=newPlaceSite&site=S000098) 119 ATATT

(-) CAATBOX1 [S000028](https://sogo.dna.affrc.go.jp/cgi-bin/sogo.cgi?sid=&pj=640&action=newPlaceSite&site=S000028) 121 CAAT

(-) CCAATBOX1 [S000030](https://sogo.dna.affrc.go.jp/cgi-bin/sogo.cgi?sid=&pj=640&action=newPlaceSite&site=S000030) 121 CCAAT

b: The predicted results of the 5’UTR of *Ghuhrf1*

ACAAAATGGCATAAATGTAAAAAAATGAATTAAAAAGAATAATATCGTCAATTCATATTTATCCTCTTCAGGAATTCAAAAAGTCTCTCAAATTCCATACCGAAGCCCCCCCGCCTCTCTCTCTCTATATATCTTTTCTCCTCTCGCCCACTCTTCCCTCCAGTCTCCGTCCCATCAAAATTCAACGTCTGCTATCTTGCGCCTCAAGCTCATTGTTTGTTTGCCA

226 base pairs

(+) = Current Strand

(-) = Opposite Strand

1 ACAAAATGGCATAAATGTAAAAAAATGAATTAAAAAGAATAATATCGTCA

(+) L1BOXATPDF1 [S000386](https://sogo.dna.affrc.go.jp/cgi-bin/sogo.cgi?sid=&pj=640&action=newPlaceSite&site=S000386) 12 TAAATGYA

(-) INRNTPSADB [S000395](https://sogo.dna.affrc.go.jp/cgi-bin/sogo.cgi?sid=&pj=640&action=newPlaceSite&site=S000395) 22 YTCANTYY

(+) POLASIG2 [S000081](https://sogo.dna.affrc.go.jp/cgi-bin/sogo.cgi?sid=&pj=640&action=newPlaceSite&site=S000081) 28 AATTAAA

(+) DOFCOREZM [S000265](https://sogo.dna.affrc.go.jp/cgi-bin/sogo.cgi?sid=&pj=640&action=newPlaceSite&site=S000265) 34 AAAG

(-) -10PEHVPSBD [S000392](https://sogo.dna.affrc.go.jp/cgi-bin/sogo.cgi?sid=&pj=640&action=newPlaceSite&site=S000392) 36 TATTCT

(+) POLASIG3 [S000088](https://sogo.dna.affrc.go.jp/cgi-bin/sogo.cgi?sid=&pj=640&action=newPlaceSite&site=S000088) 38 AATAAT

(-) ROOTMOTIFTAPOX1 [S000098](https://sogo.dna.affrc.go.jp/cgi-bin/sogo.cgi?sid=&pj=640&action=newPlaceSite&site=S000098) 41 ATATT

(-) GATABOX [S000039](https://sogo.dna.affrc.go.jp/cgi-bin/sogo.cgi?sid=&pj=640&action=newPlaceSite&site=S000039) 43 GATA

(-) ASF1MOTIFCAMV [S000024](https://sogo.dna.affrc.go.jp/cgi-bin/sogo.cgi?sid=&pj=640&action=newPlaceSite&site=S000024) 46 TGACG

(-) WBOXATNPR1 [S000390](https://sogo.dna.affrc.go.jp/cgi-bin/sogo.cgi?sid=&pj=640&action=newPlaceSite&site=S000390) 47 TTGAC

(-) WRKY71OS [S000447](https://sogo.dna.affrc.go.jp/cgi-bin/sogo.cgi?sid=&pj=640&action=newPlaceSite&site=S000447) 47 TGAC

(+) CAATBOX1 [S000028](https://sogo.dna.affrc.go.jp/cgi-bin/sogo.cgi?sid=&pj=640&action=newPlaceSite&site=S000028) 49 CAAT

51 ATTCATATTTATCCTCTTCAGGAATTCAAAAAGTCTCTCAAATTCCATAC

(+) ROOTMOTIFTAPOX1 [S000098](https://sogo.dna.affrc.go.jp/cgi-bin/sogo.cgi?sid=&pj=640&action=newPlaceSite&site=S000098) 55 ATATT

(-) GT1CONSENSUS [S000198](https://sogo.dna.affrc.go.jp/cgi-bin/sogo.cgi?sid=&pj=640&action=newPlaceSite&site=S000198) 58 GRWAAW

(-) IBOXCORE [S000199](https://sogo.dna.affrc.go.jp/cgi-bin/sogo.cgi?sid=&pj=640&action=newPlaceSite&site=S000199) 59 GATAA

(+) SREATMSD [S000470](https://sogo.dna.affrc.go.jp/cgi-bin/sogo.cgi?sid=&pj=640&action=newPlaceSite&site=S000470) 59 TTATCC

(-) GATABOX [S000039](https://sogo.dna.affrc.go.jp/cgi-bin/sogo.cgi?sid=&pj=640&action=newPlaceSite&site=S000039) 60 GATA

(-) MYBST1 [S000180](https://sogo.dna.affrc.go.jp/cgi-bin/sogo.cgi?sid=&pj=640&action=newPlaceSite&site=S000180) 60 GGATA

(+) NODCON2GM [S000462](https://sogo.dna.affrc.go.jp/cgi-bin/sogo.cgi?sid=&pj=640&action=newPlaceSite&site=S000462) 64 CTCTT

(+) OSE2ROOTNODULE [S000468](https://sogo.dna.affrc.go.jp/cgi-bin/sogo.cgi?sid=&pj=640&action=newPlaceSite&site=S000468) 64 CTCTT

(-) EECCRCAH1 [S000494](https://sogo.dna.affrc.go.jp/cgi-bin/sogo.cgi?sid=&pj=640&action=newPlaceSite&site=S000494) 71 GANTTNC

(+) ERELEE4 [S000037](https://sogo.dna.affrc.go.jp/cgi-bin/sogo.cgi?sid=&pj=640&action=newPlaceSite&site=S000037) 73 AWTTCAAA

(+) DOFCOREZM [S000265](https://sogo.dna.affrc.go.jp/cgi-bin/sogo.cgi?sid=&pj=640&action=newPlaceSite&site=S000265) 80 AAAG

(-) SURECOREATSULTR11 [S000499](https://sogo.dna.affrc.go.jp/cgi-bin/sogo.cgi?sid=&pj=640&action=newPlaceSite&site=S000499) 83 GAGAC

101 CGAAGCCCCCCCGCCTCTCTCTCTCTATATATCTTTTCTCCTCTCGCCCA

(+) CTRMCAMV35S [S000460](https://sogo.dna.affrc.go.jp/cgi-bin/sogo.cgi?sid=&pj=640&action=newPlaceSite&site=S000460) 116 TCTCTCTCT

(+) CTRMCAMV35S [S000460](https://sogo.dna.affrc.go.jp/cgi-bin/sogo.cgi?sid=&pj=640&action=newPlaceSite&site=S000460) 118 TCTCTCTCT

(-) GATABOX [S000039](https://sogo.dna.affrc.go.jp/cgi-bin/sogo.cgi?sid=&pj=640&action=newPlaceSite&site=S000039) 130 GATA

(-) NODCON1GM [S000461](https://sogo.dna.affrc.go.jp/cgi-bin/sogo.cgi?sid=&pj=640&action=newPlaceSite&site=S000461) 131 AAAGAT

(-) OSE1ROOTNODULE [S000467](https://sogo.dna.affrc.go.jp/cgi-bin/sogo.cgi?sid=&pj=640&action=newPlaceSite&site=S000467) 131 AAAGAT

(-) DOFCOREZM [S000265](https://sogo.dna.affrc.go.jp/cgi-bin/sogo.cgi?sid=&pj=640&action=newPlaceSite&site=S000265) 133 AAAG

(-) POLLEN1LELAT52 [S000245](https://sogo.dna.affrc.go.jp/cgi-bin/sogo.cgi?sid=&pj=640&action=newPlaceSite&site=S000245) 135 AGAAA

(+) CACTFTPPCA1 [S000449](https://sogo.dna.affrc.go.jp/cgi-bin/sogo.cgi?sid=&pj=640&action=newPlaceSite&site=S000449) 149 YACT

151 CTCTTCCCTCCAGTCTCCGTCCCATCAAAATTCAACGTCTGCTATCTTGC

(+) NODCON2GM [S000462](https://sogo.dna.affrc.go.jp/cgi-bin/sogo.cgi?sid=&pj=640&action=newPlaceSite&site=S000462) 151 CTCTT

(+) OSE2ROOTNODULE [S000468](https://sogo.dna.affrc.go.jp/cgi-bin/sogo.cgi?sid=&pj=640&action=newPlaceSite&site=S000468) 151 CTCTT

(-) SURECOREATSULTR11 [S000499](https://sogo.dna.affrc.go.jp/cgi-bin/sogo.cgi?sid=&pj=640&action=newPlaceSite&site=S000499) 163 GAGAC

(+) PALBOXAPC [S000137](https://sogo.dna.affrc.go.jp/cgi-bin/sogo.cgi?sid=&pj=640&action=newPlaceSite&site=S000137) 167 CCGTCC

(-) ACGTATERD1 [S000415](https://sogo.dna.affrc.go.jp/cgi-bin/sogo.cgi?sid=&pj=640&action=newPlaceSite&site=S000415) 185 ACGT

(+) ACGTATERD1 [S000415](https://sogo.dna.affrc.go.jp/cgi-bin/sogo.cgi?sid=&pj=640&action=newPlaceSite&site=S000415) 185 ACGT

(-) GATABOX [S000039](https://sogo.dna.affrc.go.jp/cgi-bin/sogo.cgi?sid=&pj=640&action=newPlaceSite&site=S000039) 193 GATA

201 GCCTCAAGCTCATTGTTTGTTTGCCA

(-) CAATBOX1 [S000028](https://sogo.dna.affrc.go.jp/cgi-bin/sogo.cgi?sid=&pj=640&action=newPlaceSite&site=S000028) 212 CAAT

(-) AACACOREOSGLUB1 [S000353](https://sogo.dna.affrc.go.jp/cgi-bin/sogo.cgi?sid=&pj=640&action=newPlaceSite&site=S000353) 215 AACAAAC

(-) ANAERO1CONSENSUS [S000477](https://sogo.dna.affrc.go.jp/cgi-bin/sogo.cgi?sid=&pj=640&action=newPlaceSite&site=S000477) 216 AAACAAA

**Figure S2** The putative cis-acting elements in the 5’UTRs of *Ghrack1* (a) and *Ghuhrf1* (b) determined by the software PLACE. These two sequences included some core elements, such as the CAAT-Box and GATA-Box, but no TATA-Box.
