# Supplementary material for: Structural and Functional Analysis of a Bidirectional Promoter from Gossypium hirsutum in Arabidopsis
Source: Int J Mol Sci. 2018 Oct 23;19(11):3291. doi: 10.3390/ijms19113291 (PMC6274729; doi:10.3390/ijms19113291)
Supplement: Supplementary file 1 [file ijms-19-03291-s001.zip › Supplementary materials/Figure S3.docx]

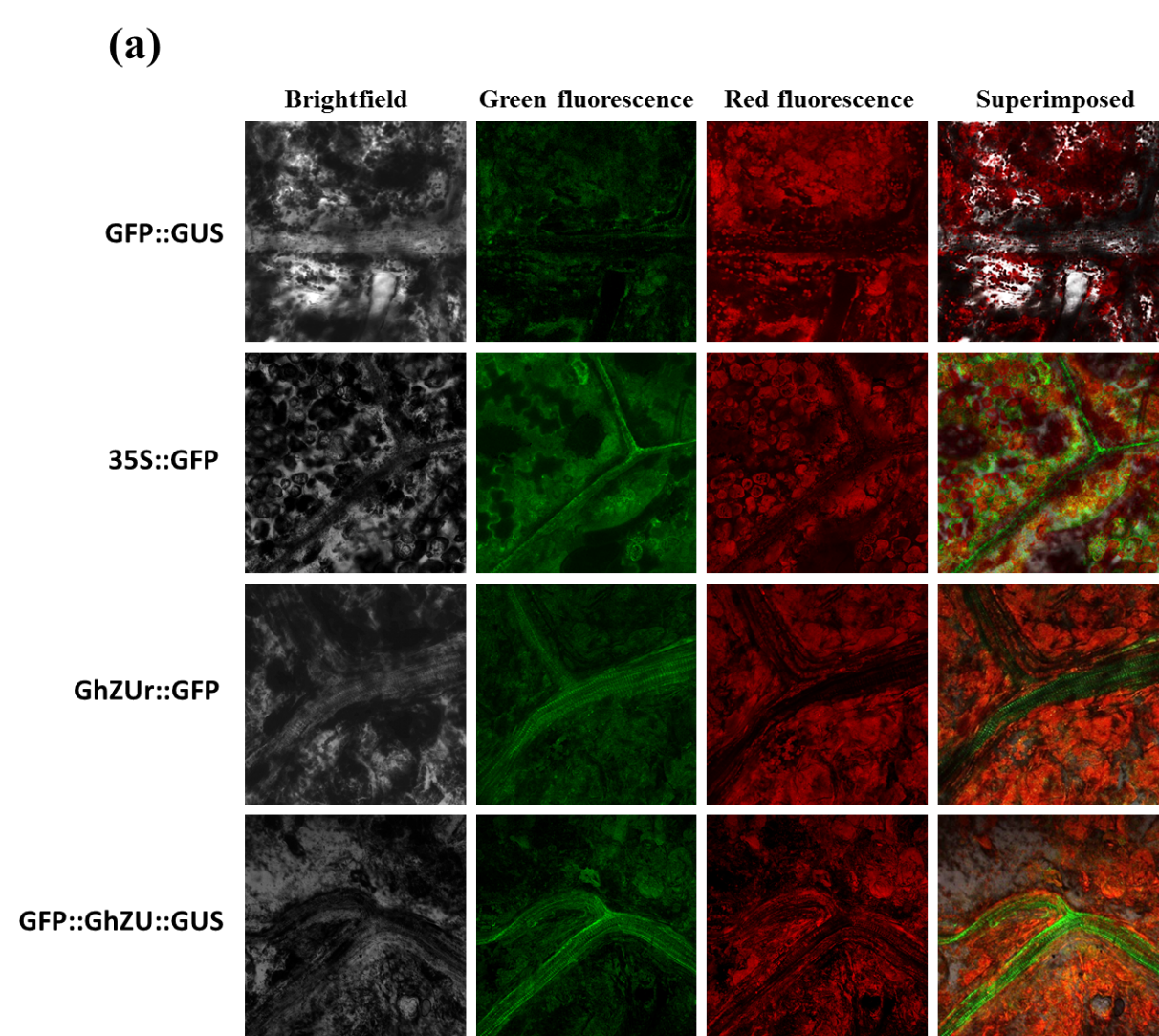


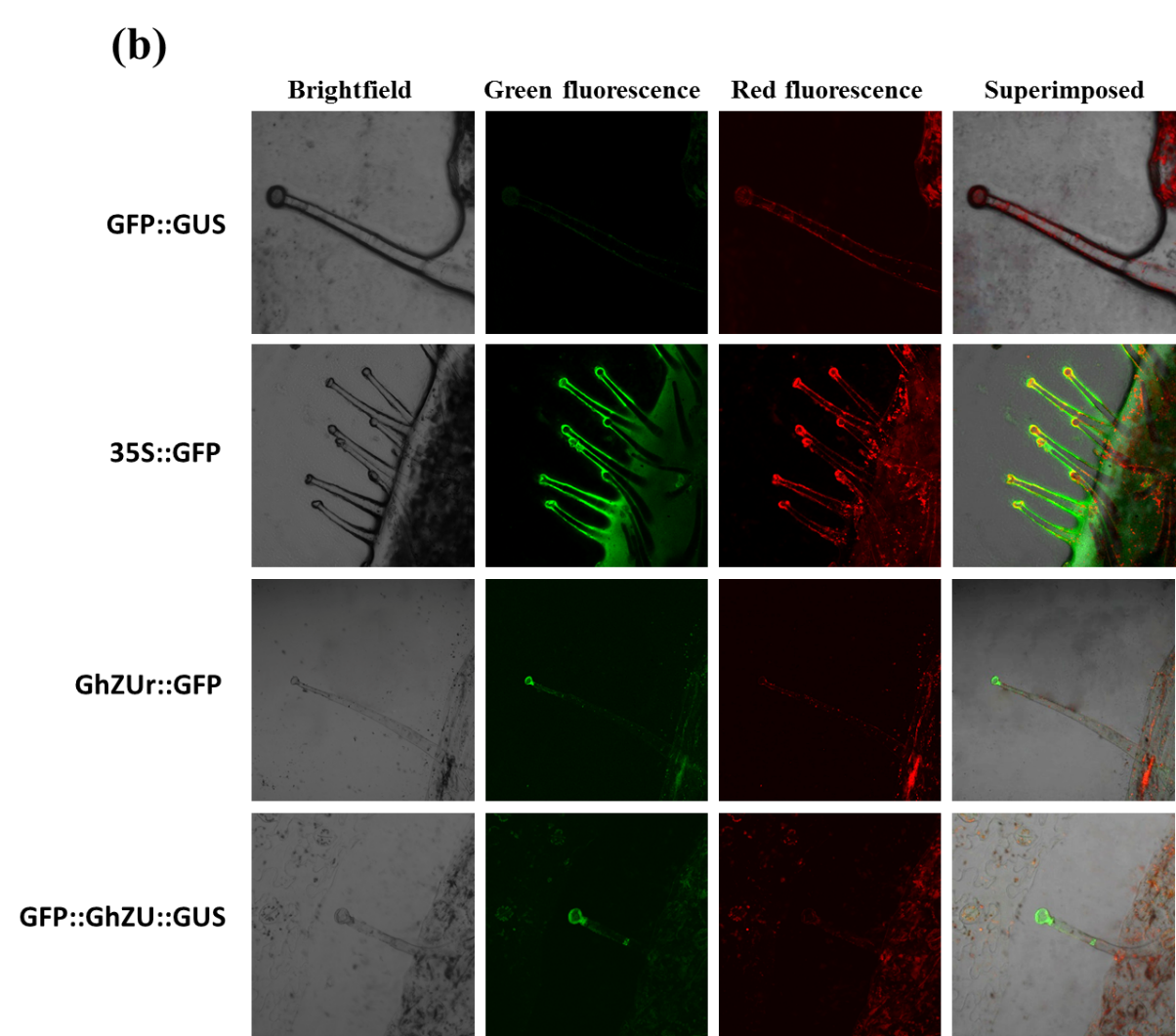


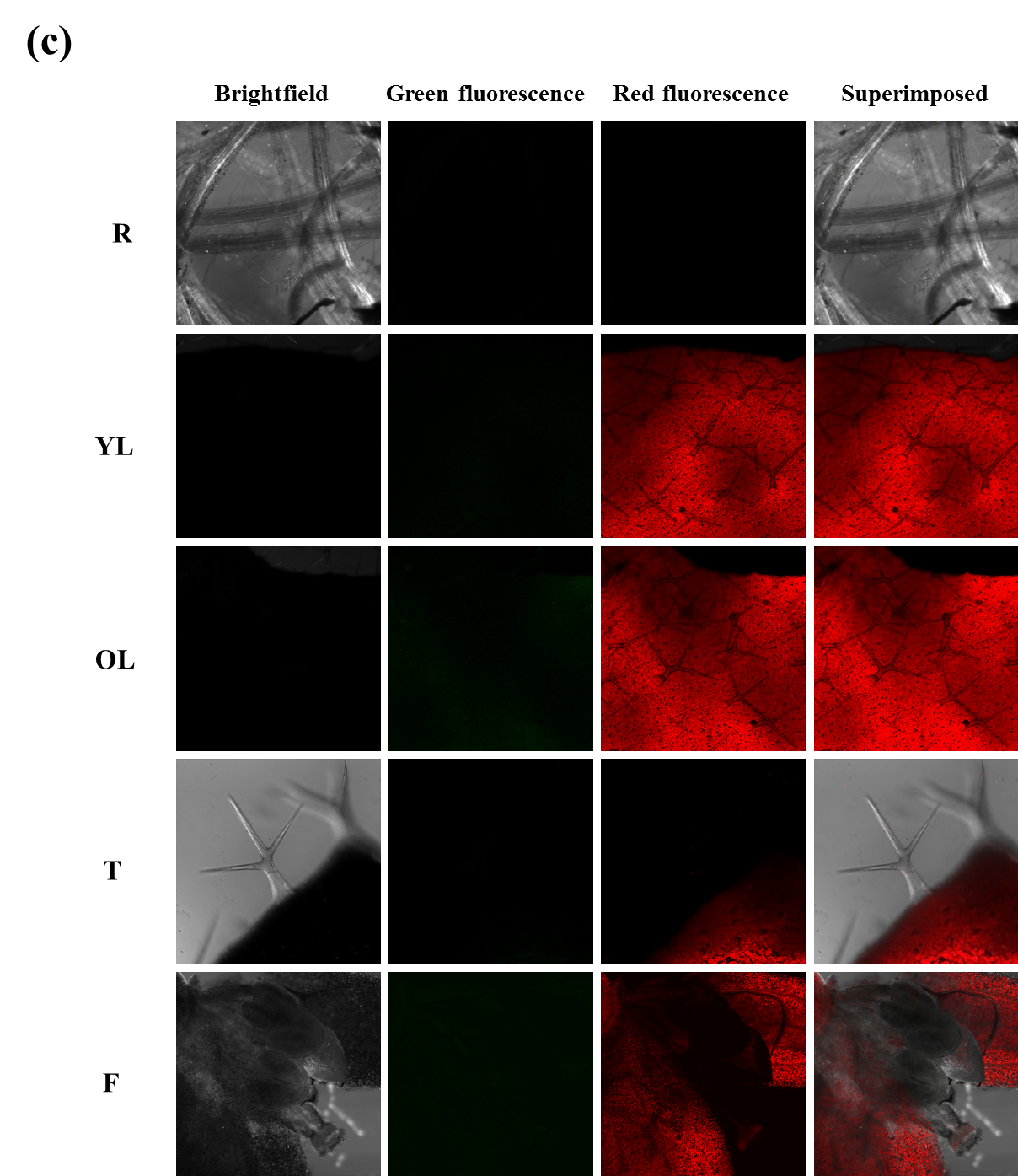


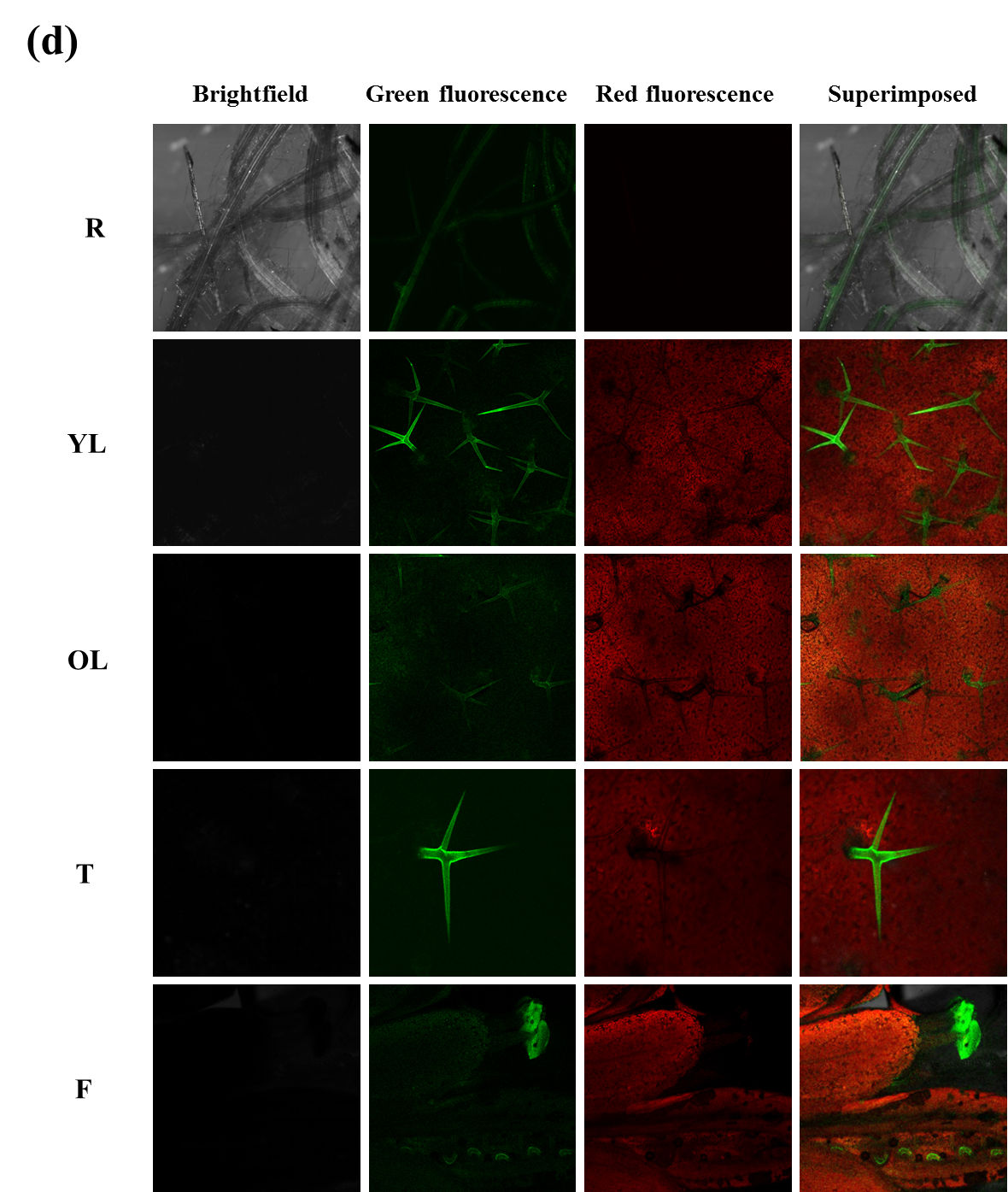


**Figure S3** Transient expression and stable expression of *gfp* gene in tobacco and transgenic *Arabidopsis*. (a-b) Transient expression of *gfp* gene in tobacco. Confocal laser scanning microscopic analysis of *gfp* expression under GFP::GUS, 35S::GFP, GhZUr::GFP and GFP::GhZU::GUS in tobacco leaf veins (a) and leaf trichomes (b). (c-d) Stable expression of *gfp* gene in transgenic *Arabidopsis*. Confocal laser scanning microscopic analysis of *gfp* expression under GFP::GUS (c) and GFP::GhZU::GUS (d) in transgenic *Arabidopsis* plants. (R) Root, (YL) young leaf trichomes, (OL) old leaf trichomes, (T) trichomes and (F) flower.
