# Supplementary material for: Structural and Functional Analysis of a Bidirectional Promoter from Gossypium hirsutum in Arabidopsis
Source: Int J Mol Sci. 2018 Oct 23;19(11):3291. doi: 10.3390/ijms19113291 (PMC6274729; doi:10.3390/ijms19113291)
Supplement: Supplementary file 1 [file ijms-19-03291-s001.zip › Supplementary materials/Figure S4.docx]

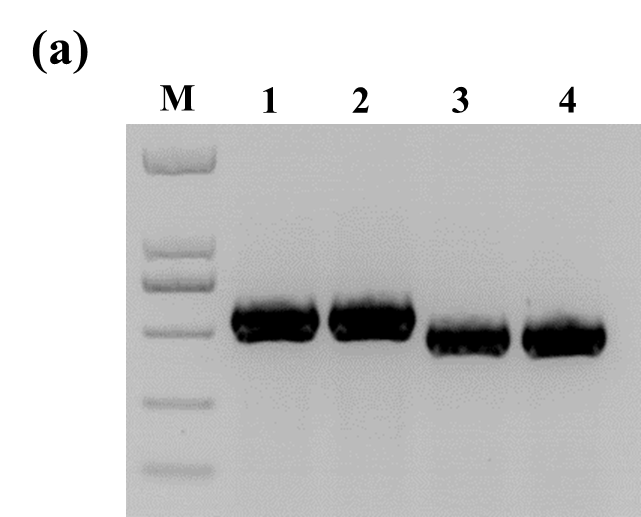


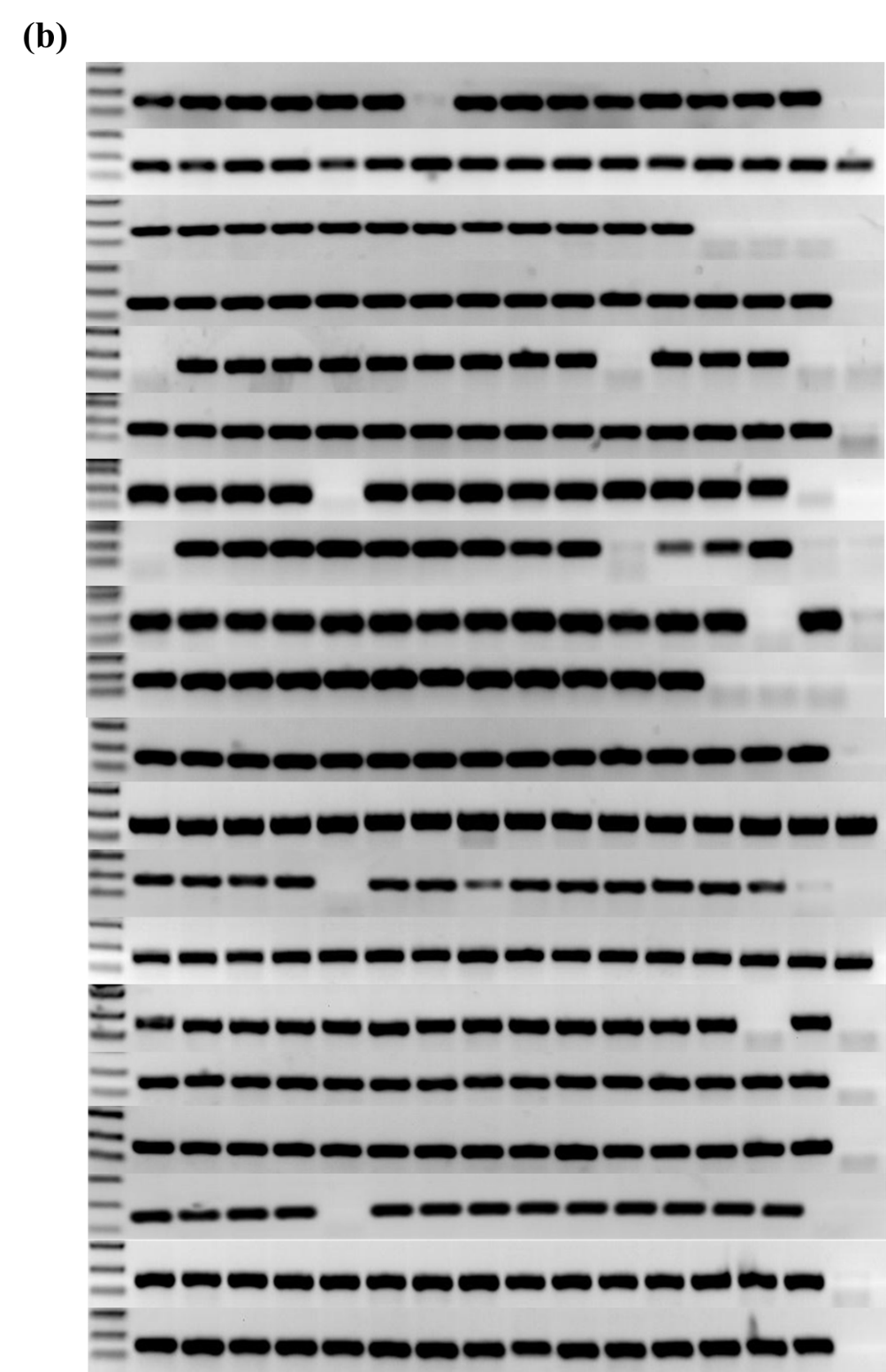


**Figure S4** Precise identification of transcription start sites (TSS) in *Ghrack1* and *Ghuhrf1* by 5'-RACE and detection of transgenic *Arabidopsis thaliana*. (a) Schematic diagram of the TSS in *Ghrack1* and *Ghuhrf1,* M: 2 kb maker, 1,2: The total length (497 bp) of 5’UTR (226 bp) plus partial sequence (271 bp) of *Ghuhrf1*. 3,4: The total length (406 bp) of 5’UTR (130 bp) plus partial sequence (276 bp) of *Ghrack1*. (b) Detection of transgenic *Arabidopsis thaliana* by PCR amplification. 246 transgenic positive plants were screened out from 320 individuals.
