# Supplementary material for: Structural and Functional Analysis of a Bidirectional Promoter from Gossypium hirsutum in Arabidopsis
Source: Int J Mol Sci. 2018 Oct 23;19(11):3291. doi: 10.3390/ijms19113291 (PMC6274729; doi:10.3390/ijms19113291)
Supplement: Supplementary file 1 [file ijms-19-03291-s001.zip › Supplementary materials/Table S1.docx]

**Table S1**. The primers used in this study

| Primer | Sequence (5'-3') |
| --- | --- |
| GeneRacer 5’ Primer | CGACTGGAGCACGAGGACACTGA |
| GeneRacer 5’ Nested Primer | GGACACTGACATGGACTGAAGGAGTA |
| Ghrack1-GSP1 | GCATCGCCCTCTTGGATAGTGT |
| Ghrack1-GSP2 | CCATAGACGAAGTTCACCATCCC |
| Ghuhrf1-GSP1 | TCACTAGTTCCTGCCTCTTCCTCGC |
| Ghuhrf1-GSP2 | CATCCGCCTCTATTGCCTTTATCGC |
| GhZU-F | TTTCGTCGGCGCTTGCGGCGG |
| GhZU-R | TGGCAAACAAACAATGAGCTTGAG |
| GhZUf-F | TACCCGGGGATCCTCTAGATTTCGTCGGCGCTTGCGGCG |
| GhZUf-R | TACCCTCAGATCTACCATGGTGGCAAACAAACAATGAGC |
| gfp-F | GTACCCGGGGATCCTCTAGATCACACGTGGTGGTGGTGG |
| gfp-R | AAGCGCCGACGAAATCTAGAATGGTAGATCTGACTAGTAAAGG |
| GhZUr-F | TCTGCTATCTTGCGCCTCAAATTTACCCTCAGATCTACCATG |
| GhZUr-R | CTAGTCAGATCTACCATTCTAGATTTCGTCGGCGCTTGCGG |
| q-Ghrack1-F | CCTAATAGGTACTGGCTTTGTG |
| q-Ghrack1-R | TTCCCCAAACTCTGATAACACC |
| q-Ghuhrf1-F | AGGCAATAGAGGCGGATGAGTC |
| q-Ghuhrf1-R | ACGCTTCCCTTGACCTATCCAC |
| q-sad-F | CCAAAGGAGGTGCCTGTTCA |
| q-sad-R | TTGAGGTGAGTCAGAATGTTGTTC |
| q-gfp-F | TGCTGAAGTCAAGTTTGAGGGAG |
| q-gfp-R | GAAGTTGGCTTTGATGCCGTTC |
| q-gus-F | TGTCATCCTCTGGGAACCACTG |
| q-gus-R | CCGTTGATAGGAGTGTCCTCATG |
| q-actin-F | GCGGTAGATTCGTTCCTCGTG |
| q-actin-R | GCTCCTTCCGTGTAGTGTCC |
| GUS-JC-F | TGTCATCCTCTGGGAACCACTG |
| GUS-JC-R | CCGTTGATAGGAGTGTCCTCATG |
| RG-F | GCGGAGCATAGGGTGATAGC |
| RG-R | TGTAACTTAGGAGCATCGAGCG |
| RG_taqman | ATGGGCCAATCAAAGATCTCCTGTCTGC |
| GUS-F | TGTAACTTAGGAGCATCGAGCG |
| GUS-R | CCGTTGATAGGAGTGTCCTCATG |
| GUS_taqman | ACGACGGACTGACCATCGATGTCTATGA |
