# Supplementary material for: Structural and Functional Analysis of a Bidirectional Promoter from Gossypium hirsutum in Arabidopsis
Source: Int J Mol Sci. 2018 Oct 23;19(11):3291. doi: 10.3390/ijms19113291 (PMC6274729; doi:10.3390/ijms19113291)
Supplement: Supplementary file 1 [file ijms-19-03291-s001.zip › Supplementary materials/Table S2.docx]

**Table S2** Putative cis-regulatory elements in the GhZU-1073 bp by PLACE and PlantCARE

|  | **Function** | **Name** | **Sequence** | **Origin** | **Position** | **Orientation** |
| --- | --- | --- | --- | --- | --- | --- |
| **1** | Transcriptional activation | GT1CONSENSUS | GRWAAW | Arabidopsis thaliana | 758, 783, 798 | + |
|  |  |  |  |  | 115, 260, 564, 611, 708, 822, 861, 1007 | - |
| **2** |  | ARR1AT | NGATT | Oryza sativa | 294, 452, 597, 938, 942 | + |
|  |  |  |  |  | 121, 150, 283, 482, 517, 807, 984, 992 | - |
| **3** | Binding with RNA transcription factor | CAAT-box | CAAT | Tobacco | 10, 120, 290, 365 | + |
|  |  |  |  |  | 443, 490, 697, 936, 940, 959 | - |
| **4** | Ethylene response | ERELEE4 | AWTTCAAA | Tobacco | 335,460 | + |
|  |  |  |  |  | 330 | - |
| **5** | ABA-responsive | DPBFCOREDCDC3 | ACACNNG | Arabidopsis thaliana | 232 | + |
| **6** | Cotton fiber development and elongation | MYB2AT | TAACTG | Tobacco | 432 | + |
|  |  | MYB2CONSENSUSAT | YAACKG | Tobacco | 432，676，1033 | + |
|  |  |  |  |  | 448，562，964 | - |
|  |  | IBOXCORE | GATAA | Cotton | 457，602 | + |
|  |  |  |  |  | 418，549，689 | - |
| **7** | Pollen specific  expression | POLLEN1LELAT52 | AGAAA | Tomato | 757,782,797 | + |
|  |  |  |  |  | 566,824 | - |
| **8** | Leaf specific  expression | CACTFTPPCA1 | YACT | rice | 54,127,225,242,272,722,830,999 | + |
|  |  |  |  |  | 92,371,661,1018,1062,1070 | - |
| **9** | Endosperm specific expression | AACACOREOSGLUB1 | AACAAAC | Oryza sativa | 248,252 | - |
|  |  | CANBNNAPA | CNAACAC | Brassica napus | 246 | - |
| **10** | Embryo specific expression | DPBFCOREDCDC3 | ACACNNG | maize | 232 | + |
