# Supplementary figures and images for: Structural and Functional Analysis of a Bidirectional Promoter from Gossypium hirsutum in Arabidopsis
Source: Int J Mol Sci. 2018 Oct 23;19(11):3291. doi: 10.3390/ijms19113291 (PMC6274729; doi:10.3390/ijms19113291)

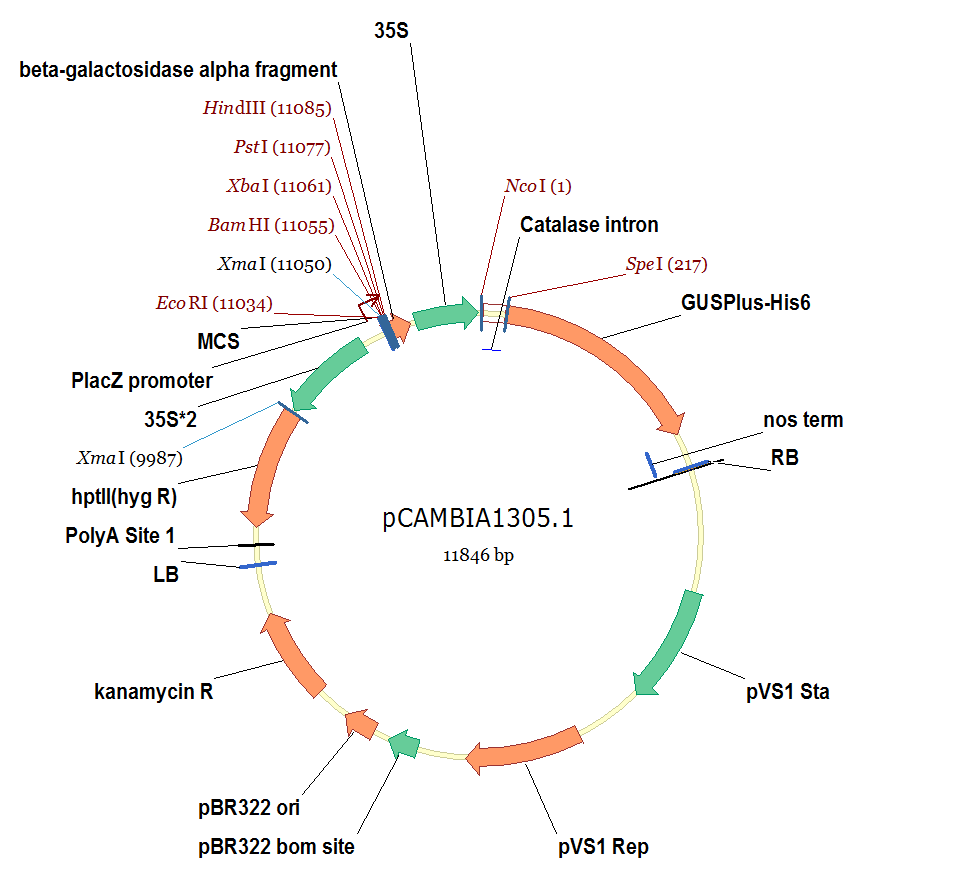


**Figure S5** The pCambia1305.1 vector

Supplement: Supplementary file 1 [file ijms-19-03291-s001.zip › Supplementary materials/Figure S5.docx]
